# Antihypertensive Drugs as Potential Repositioning Options for Melanoma: A Map of Pre-Clinical Evidence

**DOI:** 10.3390/diseases14080291

**Published:** 2026-08-12

**Authors:** Naomi Gerzvolf Mieres, Kauanna Oliveira, Nathália Carolina Barreiro Marques, Nicole Milagritos Cardoso Azorza, Helena Hiemisch Lobo Borba, Roberto Pontarolo, Marcel Henrique Marcondes Sari, Juliana Sartori Bonini, Jéssica Brandão Reolon, Raul Edison Luna Lazo, Luana Mota Ferreira

**Affiliations:** 1 Programa de Pós-Graduação em Ciências Farmacêuticas, Universidade Federal do Paraná, Curitiba 80210-170, PR, Brazil; nmgerzvolf@gmail.com (N.G.M.); kauana.oliveira@ufpr.br (K.O.); nathaliamarques@ufpr.br (N.C.B.M.); helena.borba@ufpr.br (H.H.L.B.); pontarolo@ufpr.br (R.P.); marcelsari@ufpr.br (M.H.M.S.); 2 Carrera de Farmacia y Bioquímica, Facultad de Ciencias de la Salud, Universidad Científica del Sur, Villa El Salvador, Lima 15023, Peru; 100082106@cientifica.edu.pe (N.M.C.A.); rlunal@cientifica.edu.pe (R.E.L.L.); 3 Programa de Pós-Graduação em Ciências Farmacêuticas, Departamento de Farmácia, Universidade Estadual do Centro-Oeste, Guarapuava 85040-167, PR, Brazil; jbonini@unicentro.br (J.S.B.); jessicareolon@unicentro.br (J.B.R.)

**Keywords:** melanoma therapy, antihypertensive drugs, drug repositioning, β-blockers, renin–angiotensin system, calcium channel blockers, scoping review

## Abstract

**Background/Objectives**: Melanoma remains one of the most aggressive skin cancers worldwide, with rising incidence and persistent therapeutic challenges, particularly in advanced diseases. Drug repositioning has emerged as a cost- and time-efficient strategy for identifying adjunctive treatments, and antihypertensive medications have attracted attention for their potential off-target antitumor and immunomodulatory effects. This scoping review aimed to map and critically appraise the evidence on the repositioning of antihypertensive drugs for melanoma management. **Methods**: The review followed the JBI methodology, and the results were reported in accordance with the PRISMA-ScR guidelines. Searches were conducted in July 2025 and updated in May 2026 in PubMed, Scopus, and Web of Science. For Methodological Quality and Risk-of-bias assessment, the SYRCLE Risk of Bias tool was employed for animal studies. **Results**: This review included 37 studies. β-Blockers, particularly propranolol, were the most frequently investigated agents (*n* = 21). Several studies reported antiproliferative, pro-apoptotic, antiangiogenic, and immunomodulatory effects; however, these findings were not uniform. Absent direct antiproliferative effects, biphasic dose responses, lack of angiogenic effects, and outcomes dependent on drug concentration, treatment schedule, and experimental model were also reported. Agents targeting the renin–angiotensin system and calcium channels similarly produced heterogeneous and context-dependent findings. The animal studies frequently presented high or unclear risk of bias, particularly because of incomplete reporting of randomization, allocation concealment, and blinding. No formal methodological quality assessment was performed for the in vitro studies. **Conclusions**: Antihypertensive agents, particularly β-blockers, show promising but heterogeneous preclinical signals in melanoma. However, the available evidence is not sufficient to establish reproducible efficacy or translational validity. Standardized and methodologically rigorous preclinical studies are required before these agents can be considered for clinical investigation as adjunctive melanoma therapies.

## 1. Introduction

Malignant cutaneous melanoma, one of the most aggressive types of skin cancer, develops from the uncontrolled growth of melanocytes and has a high tendency to metastasize, with an estimated global average survival rate of 69% [1,2,3,4]. Despite substantial advances in immunotherapy and targeted therapies, metastatic melanoma remains the leading cause of melanoma-related mortality. Although early diagnosis and treatment can significantly improve patient outcomes by preventing disease progression [4,5,6], current therapeutic strategies still present important limitations, including limited cellular specificity, frequent disease recurrence, treatment-related toxicity, and high costs [7,8]. Consequently, cutaneous melanoma remains the deadliest form of skin cancer and represents an attractive candidate for drug repositioning [9]. Repositioning drugs is a cost-effective strategy for developing new treatments with agents already available on the market [10]. In oncology, it favors patient access to new approaches and options, often with known safety profiles [11].

Antihypertensive drugs are among the most widespread medication classes, yet their full potential is not entirely explored in the literature. They constitute the largest group of pharmaceuticals, categorized based on their mechanisms of action: agents that target the renin–angiotensin–aldosterone system (RAAS), including angiotensin-converting enzyme inhibitors (ACEIs), drugs blocking the angiotensin type 1 receptor (AT1R), angiotensin receptor blockers (ARBs), or those directly inhibiting renin; calcium channel blockers (CCBs); adrenergic blockers that target either α- or β-adrenergic receptors; and diuretics such as thiazide, loop, and potassium-sparing diuretics, all of which reduce circulating blood volume [12,13,14]. Evidence demonstrates the potential of these agents to treat melanoma [12,15]. This interest stems from cancer-specific effects, such as aldosterone-blocking agents reducing mRNA stability and expression and preventing tumor resistance to apoptosis, and β-blockers exerting anti-angiogenic effects, possibly through downregulation of vascular endothelial growth factor (VEGF), among others [12].

While drug repositioning in oncology benefits from established safety profiles, efficacy must still be rigorously validated through clear clinical evidence or documented mechanistic pathways prior to human trials [16]. Consequently, even when researchers are permitted to bypass certain early-stage safety tests, pre-clinical studies remain essential for uncovering novel pharmacodynamic actions that were previously unexplored. This pre-clinical phase encompasses not only cellular and animal studies but also in silico approaches, which involve a suite of promising computational techniques developed to assist in drug repositioning, target identification, and the characterization of potential molecular interactions [17]. For reliable results, datasets must be accurate, comprehensive, reproducible, and representative, highlighting the importance of supplementary in vitro or in vivo assays for data validation. Nonetheless, in vivo studies should adhere to methodological reporting standards to maintain the integrity of results [18].

Given the above, this study aimed to systematically map and critically evaluate the preclinical evidence on the potential repositioning of antihypertensive drugs for melanoma treatment, encompassing both cutaneous and uveal melanoma models. Specifically, we sought to identify the pharmacological classes with the greatest translational potential, characterize the experimental models, molecular targets, and mechanistic pathways investigated, and critically appraise the methodological quality of the available evidence.

## 2. Materials and Methods

The present scoping review was conducted in accordance with the Joanna Briggs Institute (JBI) guidelines for scoping reviews, and the data were reported in accordance with the PRISMA extension for Scoping Reviews (PRISMA-ScR) [19,20]. The protocol is available on the Open Science Framework website (https://doi.org/10.17605/OSF.IO/UWN8R).

### 2.1. Eligibility Criteria

The eligibility criteria were structured according to the following guiding question: “Which antihypertensive drugs have been investigated as potential treatments for cutaneous or uveal melanoma, and what preclinical effects and mechanisms have been reported?” Original preclinical studies were eligible when they: (i) investigated cutaneous melanoma, uveal melanoma, or both, using in vitro, in vivo, or combined experimental models; (ii) evaluated a drug clinically classified as an antihypertensive agent, either alone or in combination with another intervention; and (iii) reported outcomes related to melanoma progression or antitumor activity, including cell viability, proliferation, apoptosis, migration, invasion, angiogenesis, tumor growth, metastasis, immune modulation, or survival. Studies involving other melanoma subtypes were considered eligible only when results for cutaneous or uveal melanoma models were reported separately.

Studies were excluded if they: (i) did not investigate cutaneous or uveal melanoma; (ii) did not evaluate an antihypertensive drug as an experimental intervention; (iii) did not report melanoma-related antitumor, tumor-promoting, or mechanistic outcomes; or (iv) were exclusively clinical or epidemiological and did not contain original preclinical experimental data. Reviews, editorials, conference abstracts, book chapters, and publications written in non-Roman characters were also excluded.

### 2.2. Search Strategy

The searches were conducted in July 2025 and updated in May 2026 using the PubMed, Scopus, and Web of Science databases, with terms related to melanoma, antihypertensive drugs, and their respective synonyms, combined with Boolean operators such as “OR” and “AND” (Supplementary Material—Table S1). The reference lists of the included studies were manually examined. No time restrictions were applied in the searches. All studies retrieved from the different databases were imported into Rayyan (Rayyan Systems, Inc., Doha, Qatar, free version), and all duplicates were removed [21].

### 2.3. Study Selection

In the first stage, two reviewers independently screened the titles and abstracts of all records according to the predefined eligibility criteria. Records were excluded at this stage when they clearly did not investigate cutaneous melanoma, did not evaluate an antihypertensive drug as an experimental intervention, did not report outcomes relevant to melanoma treatment, or did not constitute an original preclinical in vitro or in vivo study. Potentially eligible reports were subsequently sought for full-text retrieval and independently assessed by the same two reviewers. Disagreements during either stage were resolved by consensus or, when necessary, through consultation with a third reviewer.

### 2.4. Data Extraction and Synthesis

The data from included studies were extracted by two independent reviewers using standardized tables in Microsoft Excel^®^. Discrepancies were resolved through consensus meetings, and a third reviewer was consulted when needed. Extracted data comprised: article title, authors, year of publication, and country; drug names, type of study (in vivo or in vitro), study details (in vitro assay, cell lines, measurement, dose, administration), and main outcomes. For studies containing an in vivo component, animal sex, inclusion of both sexes, and the performance of sex-disaggregated analyses were additionally extracted.

The synthesized data were presented in a structured manner using tables, graphs, and figures to ease interpretation and comparison of results [22]. The graphical representations were created with GraphPad Prism^®^, version 8.

In addition, a visual representation of the available evidence was created using Sankey plots, which are highly effective for visualizing the distribution and relationships among variables [22,23]. These diagrams show the size and direction of connections across categories, offering clear insights into the distribution of evidence among variable combinations, such as formulations tested in specific model outcomes [22]. Additionally, Sankey plots help identify clusters where evidence is dense and gaps where some combinations are underrepresented. The data analysis and Sankey plot creation used Microsoft Excel^®^ for data organization and OpenAI tools to configure figures in the Plotly library (version 5.18.0) for dynamic visualization.

### 2.5. Quality Assessment

The risk of bias assessment was performed by two independent reviewers. The SYRCLE Tool was employed for animal studies, focusing on 6 main domains: selection, performance, detection, attrition, reporting, and other potential sources of bias, divided by 10 key questions. It includes specific signaling questions to enhance clarity, accuracy of assessment, and transparency. Each domain is classified as “Yes”, “No”, or “Unclear”, indicating a low-risk bias, a high-risk bias, or insufficient information to determine the risk, respectively [24]. The quality of in vitro studies was not evaluated due to a lack of a validated tool.

## 3. Results

### 3.1. Study Selection and Characteristics

**Figure 1:**
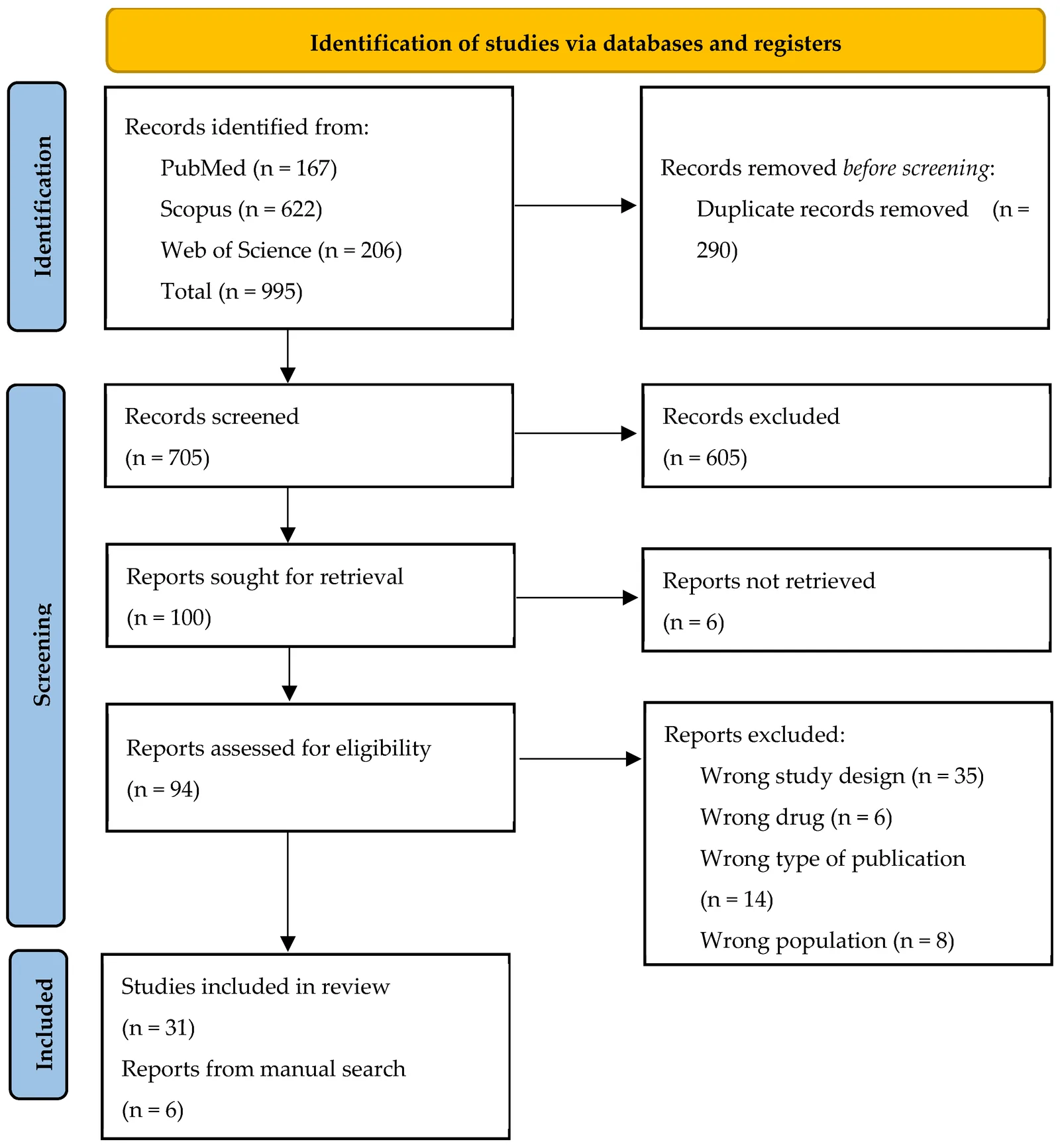
Scoping review flowchart [20].

After duplicate removal, 705 records underwent title and abstract screening. Of these, 605 were excluded for not meeting the predefined eligibility criteria, and 100 reports were sought for full-text retrieval. Six reports could not be retrieved because their full texts were unavailable through the institutional databases and other available sources. Therefore, 94 full-text reports were assessed for eligibility, of which 63 were excluded for the reasons detailed in Supplementary Table S2. Thirty-one studies were included through database searching, and six additional eligible studies were identified through manual reference-list searching, resulting in a final sample of 37 studies (Figure 1). The main data extracted are presented in Supplementary Material—Table S3.

**Figure 2:**
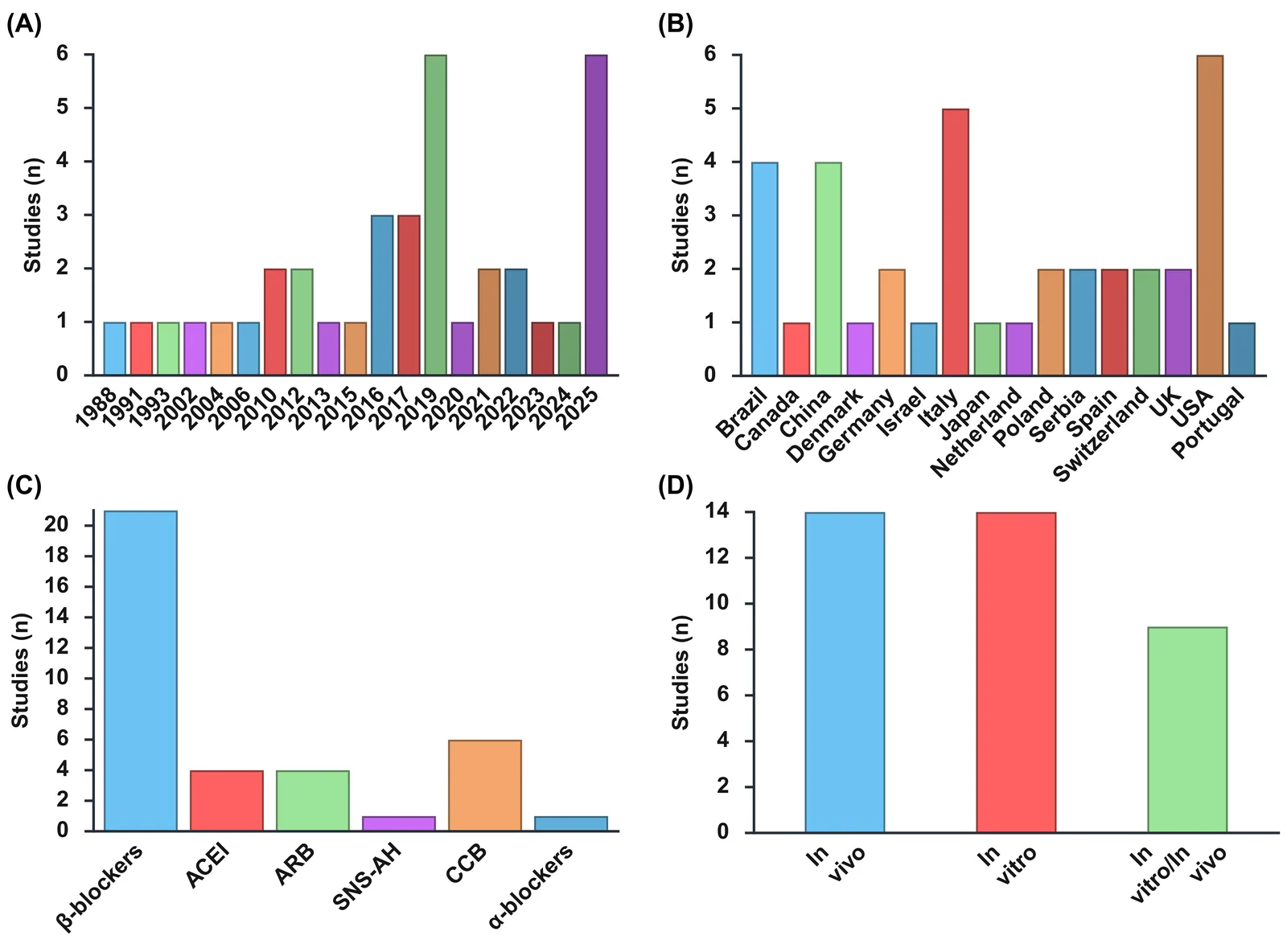
Characteristics of the articles included in the study. (**A**) Number of publications over the years; (**B**) Number of articles by country of publication; (**C**) Drug classes studied; (**D**) Study type. Note: ACEI—angiotensin-converting enzyme inhibitor; ARB—angiotensin receptor blocker; CCB—calcium channel blocker; SNS-AH—centrally and peripherally acting antiadrenergic agents.

Figure 2 comprises the descriptive data of selected studies. The 37 included studies [25,26,27,28,29,30,31,32,33,34,35,36,37,38,39,40,41,42,43,44,45,46,47,48,49,50,51,52,53,54,55,56,57,58,59,60,61] were published between 1988 and 2025, with a marked production in 2019 and 2025 (Figure 2A). In terms of geographic distribution (Figure 2B), the USA (16.22%) has the highest number of publications, followed by Italy (13.51%), Brazil (10.81%), and China (10.81%). These very distant regions underscore the global nature of repositioning drugs in oncology. Many articles are published across Western Europe, likely due to the region’s high incidence of melanoma [2].

The most studied class is β-blockers (n = 21) (Figure 2C), followed by CCBs (n = 6), and RAAS agents like ACEIs (n = 4) and ARBs (n = 4). Other classes mentioned as sympathetic nervous system non-specific acting antihypertensives (e.g., reserpine) appeared in only 1 report; another report addressed α-blockers, such as prazosin. Among the included studies, 14 involved in vivo experiments, 14 were in vitro, and 9 conducted both in vitro and in vivo studies (Figure 2D). From the 23 studies containing an in vivo protocol, nine (39.1%) used only female animals and nine (39.1%) used only male animals. Only one study (4.3%) included both male and female animals, whereas the sex of the animals was not reported in four studies (17.4%). The study, which included both sexes, pooled males and females in the statistical analyses and did not report sex-disaggregated outcomes. Consequently, none of the included studies evaluated potential sex-related differences in the efficacy or safety of the investigated interventions. Most included studies investigated cutaneous melanoma models. Three studies included uveal melanoma models: Bustamante et al. evaluated both cutaneous and uveal melanoma cell lines; Shaughnessy et al. compared the effects of calcium-channel blockade in cutaneous and uveal melanoma models; and He et al. investigated α-adrenoceptor antagonists exclusively in uveal melanoma spheroids [25,55,58].

### 3.2. Distribution of Available Evidence and Research Gaps

**Figure 3:**
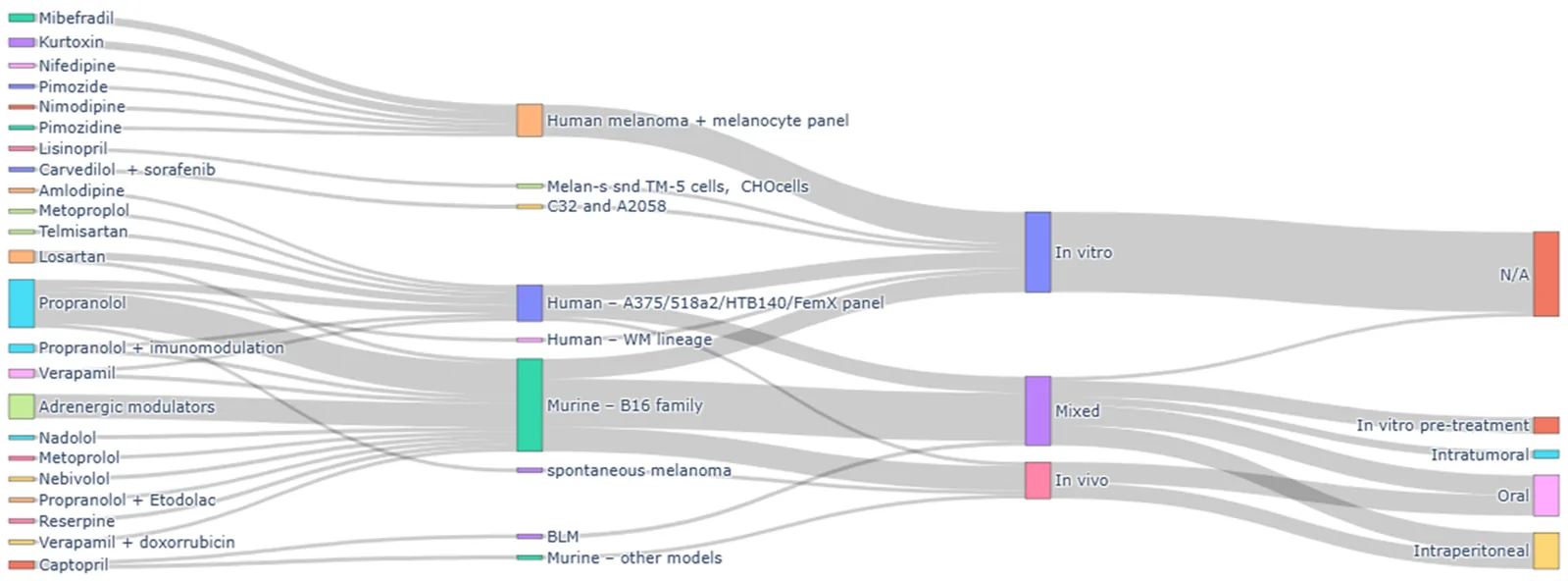
Sankey diagram on antihypertensive drugs in melanoma research therapy. The width of each flow is proportional to the number of studies represented by that connection, showing the relationships between drug classes/individual agents, experimental models, study settings, and administration routes. N/A: not applicable.

The Sankey diagram illustrates the distribution of antihypertensive drugs investigated in melanoma research (Figure 3), linking pharmacological classes and individual agents to experimental models, study settings, and routes of administration. Propranolol predominated both as monotherapy and in combination strategies, including combinations with sorafenib, etodolac, and immunomodulatory interventions, making it the most extensively investigated β-blocker in melanoma models.

Regarding biological models, most studies used murine B16 melanoma models, followed by human melanoma cell lines such as A375 and WM-derived cells. A smaller proportion investigated spontaneous melanoma or alternative murine models. This distribution highlights the strong reliance on selected animal models and human cell lines to evaluate antitumor activity and underlying mechanisms.

The included studies comprised in vitro, in vivo, and mixed designs integrating cellular assays with animal validation. Routes of administration varied across the in vivo studies, with oral and intraperitoneal administration being the most frequently employed. In some studies, melanoma cells were treated with the investigated drug before their inoculation into animals, rather than the drug being administered directly to tumor-bearing animals. These experiments were therefore classified as in vitro pretreatment followed by in vivo tumor-cell inoculation. For exclusively in vitro studies, the route of administration was recorded as “not applicable” (N/A).

Most included studies investigated cutaneous melanoma, whereas only three included uveal melanoma models. Because cutaneous and uveal melanomas have distinct biological, molecular, and clinical characteristics, findings obtained from these models should be interpreted separately rather than considered directly interchangeable. In particular, effects observed exclusively in uveal melanoma cell lines or spheroids cannot be extrapolated to cutaneous melanoma without subtype-specific validation.

From a pharmacological perspective, several factors warrant careful consideration when interpreting the translational potential of antihypertensive agents in melanoma. First, dose–response relationships observed in vitro frequently involved concentrations that may exceed clinically achievable or safe systemic exposures, raising pharmacokinetic and pharmacodynamic concerns [62]. Second, drug selectivity and receptor-affinity profiles differ substantially among pharmacological classes and even within the same class. For example, non-selective and β_1_-selective β-blockers may produce different biological effects and should not be assumed to have equivalent antitumor activity. Third, tissue distribution, blood–brain barrier permeability, and metabolic pathways may influence therapeutic activity, particularly in metastatic melanoma or central nervous system involvement [63].

The tumor microenvironment and immune modulation represent additional pharmacodynamic dimensions. Blockade of β-adrenergic signaling has been associated with reduced angiogenesis, altered cytokine expression, and enhanced natural killer cell activity, suggesting indirect antitumor mechanisms beyond direct cytotoxicity [64,65]. Conversely, agents targeting the renin–angiotensin system may exert dual or context-dependent effects through vascular remodeling and inflammatory pathways. These pleiotropic mechanisms underscore the importance of incorporating biomarker-driven analyses, receptor-expression profiling, and immune-oncological endpoints into future studies [66].

Another important limitation affecting the translational value of the available preclinical evidence is the insufficient consideration of sex as a biological variable. Among the 23 studies containing an in vivo component, nine (39.1%) used only female animals and nine (39.1%) used only male animals. Only one study included animals of both sexes, and males and females were pooled rather than analyzed separately. Animal sex was not reported in the remaining four studies (17.4%). Thus, the apparent numerical balance between male-only and female-only studies does not permit the assessment of sex-by-treatment interactions because the sexes were investigated in different experimental settings involving different drugs, melanoma models, doses, and outcomes.

This limitation is particularly relevant because melanoma incidence, progression, and prognosis differ between males and females [67]. Sex differences are also observed in the prevalence and control of hypertension and may influence the selection, efficacy, and tolerability of antihypertensive drugs [68,69]. Furthermore, sex has been investigated as a potential modifier of responses to immune checkpoint inhibitors. Although an initial meta-analysis suggested a greater overall survival benefit among male patients [70], subsequent and more comprehensive analyses found that immune checkpoint inhibitors improved overall and progression-free survival in both sexes, without a statistically significant sex-by-treatment interaction [71,72]. These findings further emphasize the need to investigate sex as a biological variable rather than assume equivalent treatment effects. Consequently, the current preclinical evidence cannot establish whether the antitumor and immunomodulatory effects of antihypertensive agents are comparable between sexes. Future studies should include adequately powered groups of both sexes, prespecify sex as an experimental factor, test sex-by-treatment interactions, and report sex-disaggregated efficacy and safety outcomes.

Although propranolol was the most frequently investigated agent, its effects were not uniform across the included studies. Antiproliferative, pro-apoptotic, antiangiogenic, and immunomodulatory activities were reported in several experimental settings. Nevertheless, absent direct antiproliferative effects, biphasic dose responses, no detectable effects on angiogenesis, and model-dependent outcomes were also reported. Differences in concentration, dose, route of administration, treatment duration, melanoma model, and combination strategy may partly explain this variability.

Moreover, frequent high or unclear risk-of-bias judgments among animal studies reduce confidence in the reproducibility of the reported effects. The predominance of unclear risk-of-bias ratings mainly reflects insufficient reporting of methodological procedures rather than definitive evidence of inadequate study conduct. Because no formal methodological quality assessment was conducted for the in vitro studies, the internal validity and overall certainty of this portion of the evidence could not be formally determined. The findings should therefore be interpreted as promising and hypothesis-generating rather than as evidence of reproducible or translationally validated efficacy.

Drug–drug interactions and combination strategies also require systematic evaluation because antihypertensive agents may be administered alongside immunotherapies or targeted therapies. Pharmacokinetic interactions, metabolic enzyme inhibition, and synergistic or antagonistic modulation of signaling pathways may enhance or attenuate therapeutic efficacy. Overall, although the pharmacological rationale for repositioning antihypertensive agents in melanoma is biologically plausible, robust translational validation requires clinically relevant exposure parameters, standardized outcome measures, and methodologically rigorous experimental designs.

### 3.3. Assessment of Bias for Animal Studies

**Figure 4:**
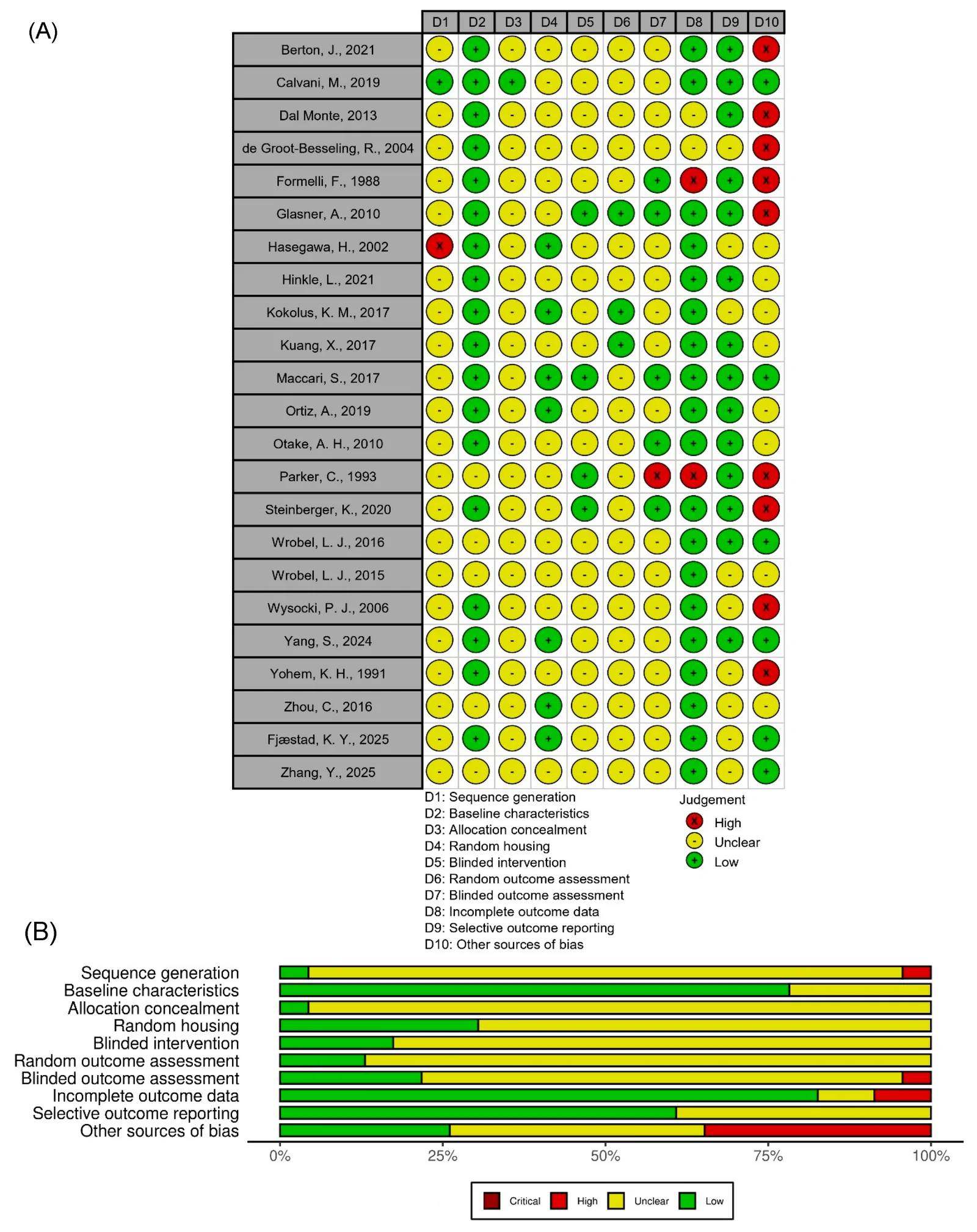
Summary of risk of bias evaluation for included studies (SYRCLE tool). (**A**) = Traffic light visualization, (**B**) = summary view [74].

The risk of bias assessment using the SYRCLE tool revealed that all studies had the majority of domains classified as having an unclear risk of bias due to insufficient methodological reporting; 10 articles had at least one domain classified as having a high risk of bias (43.5%) (Figure 4A). It should be emphasized that an unclear risk-of-bias judgment does not necessarily indicate poor methodological quality, but rather that the available information was insufficient to determine whether appropriate measures had been implemented. These findings highlight important reporting deficiencies, particularly regarding randomization, blinding, and allocation concealment (Figure 4B), underscoring the need for improved reporting and methodological standardization in this type of research.

A critical appraisal of the available evidence reveals that, although the volume of studies investigating antihypertensive drugs in melanoma has increased, methodological heterogeneity and variable study quality remain substantial limitations. Many investigations employ different cell lines, dosing regimens, exposure times, and outcome measures, complicating cross-study comparability and limiting reproducibility. In animal models, incomplete reporting of randomization procedures, blinding, and allocation concealment is frequently observed, introducing potential bias [18]. As a result, many SYRCLE domains were classified as presenting an unclear risk of bias. Clearly reporting methods for sequence generation and allocation concealment remain uncommon in in vivo studies, preventing a robust methodological appraisal. Blinding during intervention and outcome assessment is also not routinely described, despite the recommendations of the Animal Research: Reporting of In Vivo Experiments (ARRIVE) guidelines, which require authors to specify who performed the analyses and how they were conducted. Only a few reports fully meet these criteria, whereas others rely on ambiguous terminology, making methodological assessment difficult [18,73].

## 4. Discussion

### 4.1. Adrenergic Blockers

β-blockers, the most frequently mentioned class, can modulate cancer progression through multiple pathways and mechanisms (Table 1). Propranolol, a non- selective β-blocker, reduces tumor growth and volume across multiple in vitro and in vivo models [25,26,31,32,33,34,35,36,37,38,39,46,47,49]. It exerts anti-angiogenic effects by dose-dependently reducing VEGF and shows anti-proliferative and cell-cycle arrest activity through modulation of the cyclic AMP (cAMP) pathway [25,29,36,39,41,47]. Propranolol also induces apoptosis and morphological alterations, reduces migration, and inhibits stress-induced tumor progression [25,29,31,36,37,39,41,47,57,60]. Mechanistic evidence indicates that its anti-tumor effects are primarily mediated by β_2_-adrenergic receptor (β_2_-AR) blockade, rather than β_1_, with a particular emphasis on β_2_-AR involvement in immunotherapy response and cAMP-driven proliferation [33,36]. Metoprolol appears in a couple of studies [33,46,57], mostly reinforcing that the benefit of β-blockade was nonspecific or rather β_2_-specific, not demonstrating any benefits on its own, as with other β-blockers such as atenolol, isoproterenol, and zenidol [57,61]. Recent clinical and translational studies further support the need to clarify the role of β-blockers, particularly propranolol, in melanoma management. A 2025 systematic review and meta-analysis of clinical studies suggested a potential association between β-blocker use and improved disease-free survival, although evidence remains insufficient to support clinical recommendation for cancer treatment, including melanoma [75]. Therefore, despite increasing interest and ongoing clinical investigations, further prospective studies are required to establish the therapeutic relevance of β-adrenergic blockade in melanoma.

Carvedilol demonstrated antiproliferative effects and synergized with immunomodulatory therapies, sharing key targets (signal transducer and activator of transcription 3 (STAT3), hypoxia-inducible factor 1 alpha (HIF1A), mitogen-activated protein kinase family members MAPK1, MAPK3, and MAPK14 (MAPK1/3/14), myeloid cell leukemia-1 (MCL-1)) implicated in proliferation, apoptosis, and inflammation [42,57]. Nebivolol also effectively reduced migration, invasion, and proliferation of the B16 and A2058 cell lines and induced cycle arrest. In vivo, it reduced tumor volume and weight and showed reductions in metastasis and in angiogenesis markers [44]. Another adrenergic modulator, clonidine, an α_2_-adrenergic receptor (α_2_-AR) agonist, can reduce proliferation by probable crosstalk with β_2_ adrenoceptor [36]. In the same study, isoproterenol, a β-adrenergic agonist, proved that stress by catecholamines can induce tumor progression. A_1_-blockers, such as prazosin and doxazosin, can induce apoptosis in uveal melanoma spheroids [58].

Reserpine, by classification, is not an adrenergic blocker; rather, it inhibits vesicular monoamine transporter type 2 (VMAT-2), a transporter whose blockade leads to catecholamine depletion, although it was associated with tumor-derived extracellular vesicles (TEVs), which can stimulate metastasis [38]. It was found that reserpine can interfere with B16F10 TEV uptake, preventing TEV-induced downregulation of Interferon Alpha and Beta Receptor Subunit 1 (IFNAR1) and stimulating Interferon-Stimulated Genes (ISGs), and thus can be used as an adjuvant agent, as demonstrated by results showing that it virtually eliminated lung metastases and improved overall survival. However, in vitro, it did not significantly affect B16F10 cell proliferation, and as a single agent, it has a modest effect on primary tumor growth and cancer survival.

### 4.2. Renin–Angiotensin System Modulators

Evidence suggests that ARBs may have potential as adjuvant agents; however, studies involving ACEIs indicate context-dependent and dose-dependent effects, including signals of possible pro-metastatic activity, as discussed below. The ACEI lisinopril has shown conflicting effects in melanoma models, exhibiting both anti-proliferative properties and a risk of metastasis (Table 2) [27,50]. Angiotensin II (Ang-II)-mediated stimulation of angiotensin-converting enzyme (ACE) promotes melanoma cell migration, which is linked, at least in part, to reduced vinculin expression, and increases proliferation in TM-5 cells. Lisinopril attenuates this Ang-II-induced proliferation, and in silico docking supports a functional ACE-Ang-II interaction consistent with these findings [27,50]. Conversely, lisinopril also increases Matrix Metalloproteinase-2 (MMP-2) secretion in a dose-dependent manner, enhancing melanoma cell invasiveness via extracellular signal-regulated kinase 1 and -2 (ERK1/2) activation. This pro-invasive effect was blocked by the mitogen-activated protein kinase kinase (MEK) inhibitor PD98059, suggesting a potential pro-metastatic risk despite the observed anti-proliferative activity [27,50].

Losartan-treated animals showed reduced expression of angiogenesis-related genes, including Fms-like Tyrosine Kinase-1 (*Flt-1*), Fms-like Tyrosine Kinase-4 (*Flt-4*), *Tie*/*Tie-2*, thrombin receptor, cluster of Differentiation 31 (*CD31*), and Vascular Endothelial Growth Factor C (*VEGF-C*), as well as decreased microvascular density and fewer Vascular Endothelial Growth Factor Receptor 1/2 (VEGFR1/VEGFR2)-positive vascular structures. These effects were accompanied by reductions in primary tumor growth, tumor volume, and tumor weight [39]. Renziehausen et al. reported that losartan had no significant effect on the proliferation of angiotensin II receptor type 1 (AGTR1)-negative melanoma cell lines, including SKMEL224, MEL505, and SKMEL30. However, losartan blocked the angiogenic effect of AGTR1-expressing melanoma cells in an endothelial cell tube formation assay. The study also showed that AGTR1 methylation was associated with metastatic melanoma and loss of receptor expression, supporting a context-dependent role of the renin–angiotensin system in melanoma [40].

Grahovac et al. evaluated telmisartan, pioglitazone, and losartan as individual treatments in B-Raf proto-oncogene, serine/threonine kinase (BRAF)-mutated and BRAF-wild-type melanoma cell lines. Among these agents, telmisartan produced the most pronounced effects, significantly decreasing cell viability and inducing apoptosis. In A375 cells, telmisartan altered mitochondrial bioenergetics by reducing adenosine triphosphate (ATP) production and oxygen consumption, increasing relative reactive oxygen species (ROS) levels, and enhancing the phosphorylation of p38, a redox-sensitive signaling protein. The combination of telmisartan and vemurafenib also exhibited synergistic cytotoxicity against BRAF-mutant melanoma cells [30].

This aligns with evidence that AGTR1 functions as a ligand-independent tumor suppressor, and its inhibition (by losartan) may confer growth-factor independence in melanoma cells [30,39,40]. AGTR1 methylation correlates with metastatic melanoma and loss of expression [40]. More recently, Vukadinović et al. investigated newly synthesized telmisartan-amino acid conjugates, which exhibited increased intracellular accumulation, mitochondrial disruption and depolarization, and reduced interaction with AT1R while retaining cytotoxic activity against melanoma cells [59]. The combination with vemurafenib showed synergistic cytotoxicity and would therefore be beneficial as coadjuvant therapy [30].

### 4.3. Calcium Channel Blockers (CCBs)

Verapamil modulates chemoresistance dynamics and also affects proliferation and apoptosis in melanoma cell lines [43,51,56]. Mechanistic studies link the effects of CCBs to T-type channels, which were linked to melanoma viability. Other CCBs, such as mibefradil and pimozide, also showed effects on tumor progression, inducing apoptosis [52,53]. Amlodipine showed no effect on cutaneous melanoma cell lines but promising inhibition on uveal cell lines [55].

## 5. Conclusions

The available evidence identifies promising preclinical signals for selected antihypertensive agents, particularly β-adrenergic blockers, in melanoma. Several studies reported antiproliferative, pro-apoptotic, antiangiogenic, and immunomodulatory effects; however, these outcomes were not uniform and varied according to drug concentration, dose, treatment schedule, experimental model, and combination strategy. Consequently, the current evidence does not establish reproducible antitumor efficacy or translational validity.

Confidence in these findings is further limited by the frequent high or unclear risk of bias among animal studies, incomplete methodological reporting, and the absence of a formal quality assessment for the in vitro evidence. Most studies investigated cutaneous melanoma, whereas uveal melanoma was evaluated in only a small number of studies. Given the biological differences between these subtypes, findings should not be extrapolated between them without subtype-specific validation.

Further studies should employ standardized and methodologically rigorous designs, clinically relevant exposure levels, adequately powered groups of both sexes, sex-disaggregated analyses, and appropriate randomization and blinding procedures. In addition, biomarker-guided patient selection should be considered in future clinical investigations to identify melanoma subgroups more likely to benefit from antihypertensive-based strategies and to improve treatment personalization. These measures are necessary to determine separately for cutaneous and uveal melanoma whether the observed signals are reproducible and sufficiently robust to support subsequent clinical investigation.

## Figures and Tables

**Table 1:** Evidence on β-Blockers and related adrenergic agents in melanoma models.

Ref.	Drug	Cell Lines/Concentration	Animal Model	Administration Route/Dose	Main Outcomes
Wawszczyk, 2023 [42]	Carvedilol Sorafenib	C32, and A2058 0.1–10 μM	N/A		Carvedilol inhibits growth in both melanoma cell lines at ≥5 µM.
Steinberger, 2020 [41]	Nadolol Norepinephrine	B16F10 150–300 μM	C57BL/6J and C57BL/6J CCL2- mice	In vitro Pre-treatment (150–300 μM)	↑ CCL2 expression (macrophages and macrophage/B16F10 co-culture under norepinephrine.
Yang, 2024 [44]	Nebivolol	A2058, and B16 0–40 μM	BALB/c nude mice	Intraperitoneal 5 and 10 mg/kg	↓ migration and invasion; ↑ mitochondrial apoptosis; effectively cell cycle arrests; ↓ proliferation; ↓ tumor volume/weight. ↓ metastasis and angiogenesis markers.
Berton, 2021 [49]	Propranolol	B16F10	C57BL/6J mice	Oral 1.43 mg/kg and 5.71 mg/kg	↓ tumor growth; there was no anxious behavior in the animals, only the depressive behavior which did not improve.
Hasegawa, 2002 [31]	Propranolol	B16, and MethA	C57BL/6 and BALB/c mice	Oral 30 ppm	↓↓ stress-enhanced tumor growth; ↓ initial tumor growth rate; ↓ adrenal hypertrophy; ↓ thymic atrophy.
Bustamante, 2019 [25]	Propranolol	MEL270, MP41, OMM2.5, MP46, WM115, and WM266.4	N/A		PROP: ↓ proliferation; ↓ migration; ↓ VEGF; G1 cell-cycle arrest; ↑ apoptosis; ↑ cfDNA release (dose-dependent).
Mieres, 2025 [37]	Propranolol	L929, and B16F10 0.5–50 µg/mL	N/A		NC-PROP: ↑ local melanoma-targeted effect. PROP repositioning ↑ (topical therapy support); NC-PROP/PROP: ↓ B16-F10 viability (25–50 µg/mL).
Maccari, 2017 [35]	Propranolol Isopreterenol	B16F10 0.01–10 μM	C57BL/6 mice	Intraperitoneal 10–40 mg/kg	Biphasic dose–response (in vivo) curve; high dose: ↓ antitumor activity; no effect on angiogenesis; ↑ arteriogenic remodeling. Low-dose PROP: ↑ SVR: high-dose returned SVR to baseline.
Wrobel, 2016 [47]	Propranolol	N/A	C57Bl/6 mice	Oral	↑ time to primary tumor; ↓ primary tumor incidence; ↑ time to metastases; ↓ proliferation index; ↓ vessel density; ↓ pigmented lesion area; ↓ lesion severity scores.
Zhou, 2016 [48]	Propranolol	A375, Patient 3 (P-3), and patient 6 cell line (P-6) 25–400 μM	NOD/SCID mice and BALB/C nude mice	Intraperitoneal 2 and 10 mg/kg	↓ melanoma growth (BALB/C mice); ↓ A375 viability; G0/G1/S phase cell-cycle arrest; ↑ mitochondrial apoptosis. ↓ of AKT and MAPK signaling.
Wrobel, 2015 [46]	Propranolol Metoprolol	A375, Mewo, and Mel-CLS3 100 μM	NSG mice	Oral	↓ melanoma cell proliferation; ↑ cytotoxicity (tumor cell lines); no cytotoxicity in normal skin cells or primary melanocytes. ↓ tumor volume (in vivo); ↓ angiogenesis; ↑ apoptosis (primary & metastatic melanoma).
Kokolus, 2017 [33]	Propranolol Metoprolol ICI 118,551	B16F10	C57BL/6J mice	Intraperitoneal 10 mg/kg	PROP: ↑ efficacy of aPD-1 and IL-2 immunotherapies. Metoprolol failed to improve tumor control.
Kuang, 2017 [34]	Propranolol Sunitinib	A375, and Patient 8 cell line (P8) 25–200 µM	NOD/SCID mice	Oral 2 mg/kg	Combined treatment ↓ proliferation; G0/G1/S cell-cycle arrest; did not increase apoptosis markers; morphological changes observed.
Hinkle, 2021 [32]	Propranolol	B16-OVA	C57BL/6J mice	Intraperitoneal 10 mg/kg	Combination therapy ↓ tumor growth, ↑ intratumor CD8^+^ T cells, CD8^+^ effect memory T cells, and CD4^+^ T cells and ↓ immunosuppressive PD-L1^+^.
Glasner, 2010 [29]	Propranolol	B16-F10.9, and Lewis lung carcinoma d122 (3LL) 10^−5^–10^−7^ M	C57BL/6J mice	Oral 5 mg/kg	Combination: ↑ survival; ↓ lung metastasis. PROP: ↓ postoperative metastatic burden, stress-related immune suppression, ↑ NK, cytotoxicity; Neither treatment affected VEGF secretion.
Matarrese, 2022 [36]	Propranolol Clonidine Isoproterenol	B16F10 Propranolol: 1–10 µM All other drugs: 1 µM	N/A		Clonidine: ↓ B16F10 (α_2_-AR-mediated). PROP: does not affect proliferation directly; ↓ β_2_-AR signaling; ↑ clonidine antiproliferative effect (unmasked).
Calvani, 2019 [26]	Propranolol SR59230A (β3- antagonist)	B16F10 10 μM	C57BL/6 mice	Intraperitoneal 20 mg/kg	↑ early apoptosis; ↓ tumor volume. β- blockade modulated immune tolerance and ↓ tumor growth.
Dal Monte, 2013 [54]	Propranolol SR59230A L-748,337	B16F10 1–10 μM	C57BL/6J mice	Intratumoral 5 mg/kg	↓ proliferation with similar efficacy. Demonstrate that β_3_-adrenergic receptor blockade is effective in ↓ melanoma growth by influencing both tumor and endothelial cells.
Ortiz, 2019 [38]	Reserpine	Human monocytic leukemia THP-1, human 1205Lu melanoma, and B16F10	C57BL/6 mice	Intraperitoneal 1 mg/kg	↓ TEV uptake (mouse splenocytes, bone marrow, and THP-1 cells in vitro). ↑ post- surgical survival and ↓↓ lung metastases.
Rama, 2025 [57]	Carvedilol Propranolol Atenolol Metoprolol	A375 Atenolol and metoprolol: 3.90625–500 μM Carvedilol: 0.78125–100 Μm Propranolol: 10–250 μM	N/A		Atenolol and metoprolol showed no significant effects on A375 cell viability, carvedilol and propranolol: ↓ viability in a dose-dependent manner, with carvedilol being more cytotoxic. ↑ cytotoxicity, synergistic effects on combinations of carvedilol or metoprolol with cisplatin.
Fjæstad, 2025 [60]	Propranolol	B16F10	CCR2-DTR mice	Oral	Propranolol: ↓ lung and liver metastases in multiple murine cancer models. ↑ tumor-infiltrating CD4+ Th1 cells and reduced CCR2+ monocyte/MDSC-like populations. Propranolol-induced CD4+ T cells: ↑ cytotoxicity against tumor cells in an MHC-II-dependent manner.
Zhang, 2025 [61]	Isoproterenol Zenidolol	B16F10, A375, SK-MEL-28, MC38, HCT116, LoVo, and Jurkat T	C57BL/6 mice	Intraperitoneal Isoproterenol: 1.25 mg/kg Zenidol: 1 mg/kg	ADRB2 inhibition: ↓ melanoma growth, increased CD8+ T-cell infiltration and cytotoxicity, ↓ Tregs and M2 macrophages, ↑ survival.
He, 2025 [58]	Prazosin Doxazosin Terazosin Alfuzosin Silodosin Tamsulosin Phenoxybenzamine RS17053 Phenylephrine	92.1, Mel270, MM28, UPMD1, UPMD2, UPMM2, and UPMM3 0–200 µM	N/A		Prazosin was the most effective clinically available α_1_-blocker: ↓ viability and inducing apoptosis in uveal melanoma spheroids. Doxazosin also showed antitumor activity, whereas phenoxybenzamine had minimal effects. Prazosin ↓↓ spheroid growth, disintegration, long-term survival, and spheroid formation, especially at ≥20–40 µM depending on the cell line.

Note: N/A, Not Applicable; PROP, Propranolol; SVR, Systemic Vascular Resistance; TEV, Tumor-derived Extracellular Vesicles; ↑ (increased); ↓ (reduced); ↓↓ (eliminated); Conc, Concentration; AR, Adrenoreceptor; CCL2, C-C Motif Chemokine Ligand 2; cfDNA, Cell-Free DNA; NC-PROP, Nanocapsules-loaded Propranolol; G0/G1/S, cell cycle phases: Gap 0, Gap 1, Synthesis phase; AKT, Protein Kinase B; aPD-1, Anti-Programmed Cell Death Protein 1 Antibody; IL-2, Interleukin-2; NK, Natural Killer Cells; ADRB2, Adrenergic Receptor Beta 2.

**Table 2:** Evidence on angiotensin-converting enzyme inhibitors, angiotensin receptor blockers, and calcium channel blockers in melanoma.

Ref.	Drug	Cell Lines/Concentration	Animal Model	Administration Route/Dose	Main Outcomes
**Angiotensin-converting enzyme inhibitors**					
Becker, 2022 [50]	Lisinopril	MV3, and Huh7	N/A		↑ MMP-2 secretion (dose-dependent); ↑ invasiveness/migration.
de Alvarenga, 2016 [27]	Lisinopril	Melan-s, TM-5, and CHO-ACE 1 μM	N/A		Ang-II/ACE: ↑ melanoma cell migration; ↓ vinculin expression. Lisinopril: ↓ Ang-II-induced proliferation.
de Groot Besseling, 2004 [28]	Captopril	BLM	BALB/c mice	Oral 2 mM+ tPA (0–80 μg)	Captopril + 80 μg tPA: ↓ tumor growth. ↑ circulating angiostatin (tumor-bearing mice prior to and after tPA injections; however, nontumor mice.
Wysocki, 2006 [45]	Captopril	RenCa, MethA, and B78-H1	BALB/c, C57BL/6, C.B-17 scid/ScidF1 mice	Oral 25 or 60 mg/kg	No enhancement of tumor growth (melanoma model); effects limited to immunogenic tumors, not melanoma.
**Angiotensin Receptor blockers**					
Otake, 2010 [39]	Losartan	B16F10	C57BL/6 mice	Oral 75–300 mg/kg	↓ inflammatory mediators; ↓ VEGF-C; ↓ tumor growth rate; ↓ microvascular density; ↓ tumor volume/weight; ↓ VEGFR1/2^+^ vascular structures; ↓ metastatic foci size; and pulmonary metastasis count unchanged.
Renziehausen, 2019 [40]	Losartan	PMWK, C8161, SKMEL (14, 23, 224, 30), A375, WM35, WM266.4, M5, MEL505, M78, M80, and M88 100 μM	N/A		No effect on AGTR1-negative cell lines (SKMEL224, SKMEL30, MEL505) and blocked angiogenesis-inducing effect in endothelial-cell tube; AGTR1 methylation correlates with metastatic melanoma and loss of expression.
Grahovac, 2019 [30]	Telmisartan Losartan Vemurafenib	A375, 518A, HTB140, and FemX-1 100 μM; Vem/TEL 10 + 50 µM	N/A		TEL: ↓ cell viability; ↑ apoptosis; mitochondrial dysfunction (A375); ↑ p-p38. TEL + vemurafenib: ↑ synergistic cytotoxicity.
Vukadinović, 2025 [59]	Telmisartan-amino acid conjugates	A375, A375R, 518A2, HaCaT, and MRC-5	N/A		↑ intracellular accumulation; mitochondrial disruption and depolarization; ↓ AT1R interaction while retaining anticancer activity.
**Calcium channel blockers**					
Yohem, 1991 [43]	Verapamil	A375M, and C8161 12.5–125 μM	Athymic nude mice	Pre-treatment 12.5–125 μM	↓↓ metastasis; ↓ invasion (dose-dependent); ↓↓ lung metastases (~98%); ↓ migration (less pronounced); ↓ cytoskeletal organization / motility; ↓ type IV collagenase mRNA; ↓ gelatinase activity; ↓ platelet aggregation/ tumor-host interactions.
Shaughnessy, 2019 [55]	Amlodipine	MP41, Mel-270, Mel-202, OMM2.3, CHL-1, MeWo, SK-MEL-2, SK-MEL-119, Mel JuSo, IPC-298, UACC-903, SK-MEL-28, MGH-CH-1, and A375 5, 10 or 20 µM	NA		No evidence of significant growth inhibition compared to the DMSO control. But significantly and selectively ↓↓ UMM growth when compared to CMM.
Das, 2012 [52]	Nimodipine Nifedipine Kurtoxin Mibefradil Pimozidine	M9, M16, M17, M28, M29, M36, JG, and HEMn-LP	NA		VGCCs (Cav1, Cav2, Cav3) expressed in melanoma T-type channels (Cav3) enriched in melanoma vs. melanocytes and mediate Ca^2+^ influx. ↓ proliferation & ↓ viability.
Das, 2013 [53]	Mibefradil Pimozide Kurtoxin	M16, M28, and JG	NA		↓ Cell proliferation (G1/S arrest) ↑ Apoptosis. ↓↓ of basal autophagy (↑ LC3-II, ↑ p62, no increase with bafilomycin. Gene silencing mimics drug effects, confirming T-type Ca^2+^ channels as target.
Formelli, 1988 [56]	Verapamil Doxorrubicin	B16V DX: 10 ng/mL VRP: 1 µM	C57BL/6 mice	Intraperitoneal VRP: 25 mg/kg Intravenous DX: 12 mg/kg	Combination accelerates resistance development (17 vs. 27 passages), ↓ Lung metastases in resistant line. Suggests VRP modulates drug resistance dynamics.
Parker, 1993 [51]	Verapamil MSH	B16 F1, and B16 ML8 VRP 10 µM MSH 10^−7^ M	C57BL/6J mice	In vitro pre-treatment	VRP had no significant effect on metastasis by either cell line. Both compounds ↑ melaninogenesis in melanoma cell variants.

Note: ACE, Angiotensin Converting Enzyme; Ang, Angiotensin; TEL, Telmisartan.; ↑ (increased); ↓ (reduced); ↓↓ (eliminated); Conc, Concentration; VRP, Verapamil; DX, Doxorubicin; MSH, α-Melanocyte Stimulating Hormone; tPA, Tissue Plasminogen Activator; p-p38, Phosphorylated p38 MAPK; UMM, Uveal Melanoma; CMM, Cutaneous Malignant Melanoma; VGCCs, Voltage-Gated Calcium Channels; LC3-II, Microtubule-associated Protein 1 Light Chain 3-II; p62, Sequestosome-1 Protein; N/A: Not applicable.

## Data Availability

No new data were created or analyzed in this study. Data sharing is not applicable to this article.

## Supplementary Materials

The following supporting information can be downloaded at https://www.mdpi.com/article/10.3390/diseases14080291/s1. Table S1—Search strategy; Table S2—Studies excluded after full-text assessment and reasons for exclusion; Table S3—Data extraction.
