# Supplementary material for: Antihypertensive Drugs as Potential Repositioning Options for Melanoma: A Map of Pre-Clinical Evidence
Source: Diseases. 2026 Aug 12;14(8):291. doi: 10.3390/diseases14080291 (PMC13511851; doi:10.3390/diseases14080291)
Supplement: Supplementary file 1 [file diseases-14-00291-s001.zip › Supplementary material (Tables S1 and S2).pdf]

Table S1. Search strategy.

Database	Search strategy
PubMed	((Cutaneous Malignant Melanoma[Title/Abstract]) OR (melanoma[Title/Abstract]) OR (familial melanoma*[Title/Abstract]) OR (Familial Atypical Mole-Malignant Melanoma Syndrome[Title/Abstract]) OR (Dysplastic Nevus Syndrome, Hereditary[Title/Abstract]) OR (melanoma, familial[Title/Abstract]) OR (melanoma cell[Title/Abstract]) OR (melanoma cell line[Title/Abstract])) AND ((Anti-Hypertensive*[Title/Abstract]) OR (Anti Hypertensive*[Title/Abstract]) OR (Antihypertensive*[Title/Abstract]) OR (Diuretic, Osmotic[Title/Abstract]) OR (Diuretic, Potassium Sparing[Title/Abstract]) OR (Epithelial Sodium Channel blocker[Title/Abstract]) OR (Mineralocorticoid Receptor Antagonists[Title/Abstract]) OR (Sodium Chloride Symporter Inhibitors[Title/Abstract]) OR (Sodium Potassium Chloride Symporter Inhibitors[Title/Abstract]) OR (Adrenergic alpha Antagonists[Title/Abstract]) OR (alpha-Adrenergic Antagonist*[Title/Abstract]) OR (alpha Adrenergic Antagonist*[Title/Abstract]) OR (α-blocker[Title/Abstract]) OR (alpha-blocker[Title/Abstract]) OR (Adrenergic alpha-Blockers[Title/Abstract]) OR (Adrenergic alpha Blockers[Title/Abstract]) OR (alpha-Adrenergic Blockers[Title/Abstract]) OR (alpha Adrenergic Blockers[Title/Abstract]) OR (α-Adrenergic Blockers[Title/Abstract]) OR (beta-Antagonist, Adrenergic[Title/Abstract]) OR (β-blocker[Title/Abstract]) OR (β blocker[Title/Abstract]) OR (βblocker[Title/Abstract]) OR (beta-Adrenergic Antagonist[Title/Abstract]) OR (beta Adrenergic Antagonist[Title/Abstract]) OR (beta-Adrenergic Blockers[Title/Abstract]) OR (beta Adrenergic Blockers[Title/Abstract]) OR (βAdrenergic Blockers[Title/Abstract]) OR (β-Adrenergic Blockers[Title/Abstract]) OR (β Adrenergic Blockers[Title/Abstract]) OR (Angiotensin Converting Enzyme Inhibitors[Title/Abstract]) OR (ACE Inhibitor*[Title/Abstract]) OR (Kininase II Antagonists[Title/Abstract]) OR (Angiotensin II Receptor Antagonist[Title/Abstract]) OR (Angiotensin II Receptor Blocker[Title/Abstract]) OR (Calcium Channel Blocking Drug[Title/Abstract]) OR (Calcium Channel Antagonist[Title/Abstract]) OR (thiazide diuretics[Title/Abstract]) OR (Potassium Depleting Diuretics[Title/Abstract]) OR (Sodium Chloride Cotransporter Inhibitors[Title/Abstract]) OR (Thiazide Sensitive NaCl Cotransporter Inhibitors[Title/Abstract]) OR (Blockers, Ganglionic[Title/Abstract]) OR (Ganglioplegic Agent*[Title/Abstract]))

**Scopus**	(TITLE-ABS-KEY("anti-hypertensive" OR "anti hypertensive" OR "antihypertensive") OR TITLE-ABS-KEY("diuretic, osmotic" OR "diuretic, potassium sparing" OR "epithelial sodium channel blocker") OR TITLE-ABS-KEY("mineralocorticoid receptor antagonists" OR "sodium chloride symporter inhibitors") OR TITLE-ABS-KEY("sodium potassium chloride symporter inhibitors") OR TITLE-ABS-KEY("adrenergic alpha antagonist" OR "alpha-adrenergic antagonist*") OR TITLE-ABS-KEY("alpha adrenergic antagonist*") OR TITLE-ABS-KEY("α-blocker, adrenergic" OR "α-adrenergic blocker*") OR TITLE-ABS-KEY("adrenergic alpha-blockers" OR "adrenergic alpha blockers") OR TITLE-ABS-KEY("alpha-adrenergic blockers" OR "alpha adrenergic blockers") OR TITLE-ABS-KEY("beta-Antagonist, Adrenergic") OR TITLE-ABS-KEY("βblocker" OR "β blocker" OR "β-blocker") OR TITLE-ABS-KEY("beta Adrenergic Antagonist" OR "beta-Adrenergic Blockers" OR "beta Adrenergic Blockers") OR TITLE-ABS-KEY("βAdrenergic Blockers" OR "β-Adrenergic Blockers" OR "β Adrenergic Blockers") OR TITLE-ABS-KEY("Angiotensin Converting Enzyme Inhibitors" OR "ACE Inhibitor" OR "Kininase II Antagonists" OR "Angiotensin II Receptor Antagonist" OR "Angiotensin II Receptor Blocker") OR TITLE-ABS-KEY("Calcium Channel Blocking Drug" OR "Calcium Channel Antagonist") OR TITLE-ABS-KEY("thiazide diuretics" OR "Potassium Depleting Diuretics" OR "Sodium Chloride Cotransporter Inhibitors" OR "Thiazide Sensitive NaCl Cotransporter Inhibitors") OR TITLE-ABS-KEY("Ganglioplegic Agent" OR "Blockers, Ganglionic")) AND TITLE-ABS-KEY("Melanoma" OR "Cutaneous Malignant Melanoma" OR "Familial Melanoma" OR "Familial Atypical Mole Malignant Melanoma Syndrome" OR "Dysplastic Nevus Syndrome, Hereditary" OR "Malignant Melanoma*, Cutaneous")
**Web of Science**	TS=("Melanoma" OR "Cutaneous Malignant Melanoma" OR "Familial Melanoma" OR "Familial Atypical Mole Malignant Melanoma Syndrome" OR "Dysplastic Nevus Syndrome, Hereditary" OR "Malignant Melanoma, Cutaneous") AND TS=("anti-hypertensive" OR "anti hypertensive" OR "antihypertensive" OR "diuretic, osmotic" OR "diuretic, potassium sparing" OR "epithelial sodium channel blocker" OR "mineralocorticoid receptor antagonists" OR "sodium chloride symporter inhibitors" OR "sodium potassium chloride symporter inhibitors" OR "adrenergic alpha antagonist" OR "alpha-adrenergic antagonist*" OR "alpha adrenergic antagonist*" OR "α-blocker, adrenergic" OR "α-adrenergic blocker*" OR "adrenergic alpha-blockers" OR "adrenergic alpha blockers" OR "alpha-adrenergic blockers" OR "alpha adrenergic blockers" OR "beta-Antagonist, Adrenergic" OR "βblocker" OR "β blocker" OR "β-blocker" OR "beta

	Adrenergic Antagonist" OR "beta-Adrenergic Blockers" OR "beta Adrenergic Blockers" OR "βAdrenergic Blockers" OR "β-Adrenergic Blockers" OR "β Adrenergic Blockers" OR "Angiotensin Converting Enzyme Inhibitors" OR "ACE Inhibitor" OR "Kininase II Antagonists" OR "Angiotensin II Receptor Antagonist" OR "Angiotensin II Receptor Blocker" OR "Calcium Channel Blocking Drug" OR "Calcium Channel Antagonist" OR "thiazide diuretics" OR "Potassium Depleting Diuretics" OR "Sodium Chloride Cotransporter Inhibitors" OR "Thiazide Sensitive NaCl Cotransporter Inhibitors" OR "Ganglioplegic Agent" OR "Blockers, Ganglionic")

Table S2. Studies excluded after full-text assessment and reasons for exclusion.

AUTHORS	YEAR	TITLE	REASON OF EXCLUSION
Fan, B., Schooling, C. M., & Zhao, J. v.	2023	Genetic proxies for calcium channel blockers and cancer: a Mendelian randomization study.	Not retrieved
Akbar, S., & Alsharidah, M. S	2014	Are beta blockers new potential anticancer agents?	Wrong study design
Akbar, S., & Richards, J. R.	2018	Beta-blockers and cancer: An emerging treatment. In Beta-Blockers: Physiological, Pharmacological and Therapeutic Implications.	Wrong type of publication
Batalla-Covello, J., Ali, S., Xie, T., & Amit, M.	2022	β-Adrenergic signaling in skin cancer	Wrong study design
Braz, W. R., de Souza, M. G. M., da Silva, L. M., de Azevedo, C. B., Ribeiro, A. B., Barbosa, D. C. T., Molina, E. F., de Faria, E. H., Ciuffi, K. J., Rocha, L. A., Martins, C. H. G., Santiago, M. B., Santos, A. L. O., & Nassar, E. J.	2024	Antitumoral action of carvedilol-a repositioning study of the drug incorporated into mesoporous silica MCM-41.	Not retrieved
Chang, P. Y., Huang, W. Y., Lin, C. L., Huang, T. C., Wu, Y. Y., Chen, J. H., & Kao, C. H.	2015	Propranolol Reduces Cancer Risk: A Population-Based Cohort Study.	Wrong population
Chang, Q., Long, J., Hu, L., Chen, Z., Li, Q., & Hu, G.	2020	Drug repurposing and rediscovery: Design, synthesis and preliminary biological evaluation of 1-arylamino-3-aryloxypropan-2-ols as anti-melanoma agents.	Wrong drug
Chen, H. Y., Zhao, W., Na'ara, S., Gleber-Netto, F. O., Xie, T., Ali, S., Thompson, Z. M., Buell, J., Stafford, H., Nagarajan, P., Davies, M., Wong, M. K., Migden, M. R., Sharma, P., Myers, J. N., Gross, N. D., & Amit, M.	2023	Beta-Blocker Use Is Associated With Worse Relapse-Free Survival in Patients With Head and Neck Cancer	Wrong population

Cleveland, K. H., Yeung, S., Huang, K. M., Liang, S., Andresen, B. T., & Huang, Y.	2018	Phosphoproteome profiling provides insight into the mechanism of action for carvedilol-mediated cancer prevention	Wrong population
Cole, S. W., & Sood, A. K.	2012	Molecular pathways: Beta-adrenergic signaling in cancer	Wrong study design
Colucci, R., & Moretti, S.	2016	The role of stress and beta-adrenergic system in melanoma: current knowledge and possible therapeutic options.	Wrong study design
D., B., M., F., C., H., & M., M.	1988	Effect of calcium channel antagonists on cellular differentiation. Surgical Forum.	Not retrieved
de Giorgi, V., Gandini, S., Grazzini, M., Benemei, S., Marchionni, N., & Geppetti, P.	2014	In reply - Effect of β-adrenergic blockers and other antihypertensive drugs on the risk of melanoma recurrence and death	Wrong type of publication
de Giorgi, V., Gandini, S., Grazzini, M., Benemei, S., Marchionni, N., & Geppetti, P.	2012	B-blockers: a new and emerging treatment for melanoma	Wrong study design
de Giorgi, V., Geppetti, P., Lupi, C., & Benemei, S.	2020	The Role of β-Blockers in Melanoma.	Wrong study design
de Giorgi, V., Grazzini, M., Benemei, S., Marchionni, N., Geppetti, P., & Gandini, S.	2017	β-Blocker use and reduced disease progression in patients with thick melanoma: 8 years of follow-up	Wrong type of publication
de Giorgi, V., Grazzini, M., Gandini, S., Benemei, S., Marchionni, N., & Geppetti, P.	2011	Do β-blockers slow down melanoma progression? - Reply	Wrong type of publication
Deng, G. H., Liu, J., Zhang, J., Wang, Y., Peng, X. C., Wei, Y. Q., & Jiang, Y.	2014	Exogenous norepinephrine attenuates the efficacy of sunitinib in a mouse cancer model.	Wrong drug
Fitzgerald, P. J.	2012	Beta blockers, norepinephrine, and cancer: An epidemiological viewpoint.	Wrong study design

Fumagalli, C., Maurizi, N., Marchionni, N., & Fornasari, D.	2020	β-blockers: Their new life from hypertension to cancer and migraine.	Wrong study design
Giorgi, V. de, Grazzini, M., Gandini, S., Benemei, S., Lotti, T., Marchionni, N., Rossari, S., Gori, A., & Geppetti, P.	2011	The effect of beta-blocker treatment in patients with cutaneous melanoma.	Wrong type of publication
Globig, A. M., Zhao, S., Roginsky, J., Maltez, V. I., Guiza, J., Avina-Ochoa, N., Heeg, M., Araujo Hoffmann, F., Chaudhary, O., Wang, J., Senturk, G., Chen, D., O'Connor, C., Pfaff, S., Germain, R. N., Schalper, K. A., Emu, B., & Kaech, S. M.	2023	The β1-adrenergic receptor links sympathetic nerves to T cell exhaustion	Wrong drug
Hanssen, H.-P.	2011	Oncology: Beta-blockers for patients with melanoma?	Wrong type of publication
He, R. H., He, Y. J., Tang, Y. J., Zhou, H. H., McLeod, H. L., & Liu, J.	2016	The potential anticancer effect of beta-blockers and the genetic variations involved in the interindividual difference.	Wrong study design
Hempel, K., & Deimel, M.	1963	Effect of the dopa-decarboxylase inhibitor alpha-methyldopa on the incorporation of dopa into melanin and pyrocatecholamine. In vivo studies on melanoma mice with tritiated DOPA.	Wrong drug
Hollestein, L. M., & Nijsten, T.	2014	The association between beta-blockers and melanoma survival: Evidence of absence or absence of evidence?	Wrong type of publication
Jeyachandran, S.	2024	Beta-adrenergic blocking agents in cutaneous and oral melanoma.	Wrong type of publication
Ji, Y., Chen, S., Xiao, X., Zheng, S., & Li, K	2012	β-blockers: A novel class of antitumor agents.	Wrong study design
Kao, J., & Luu, B.	2019	Can propranolol prevent progression of melanoma?	Wrong study design

Kennedy, O. J., & Neary, M. T.	2022	Brief Communication on the Impact of β-blockers on Outcomes in Patients Receiving Cancer Immunotherapy	Wrong study design
Lemeshow, S., Sørensen, H. T., Phillips, G., Yang, E. v., Antonsen, S., Riis, A. H., Lesinski, G. B., Jackson, R., & Glaser, R.	2011	β-Blockers and survival among Danish patients with malignant melanoma: a population-based cohort study.	Wrong population
Lens, M.	2011	Do β-blockers slow down melanoma progression?	Wrong type of publication
Levi, B., Matzner, P., Goldfarb, Y., Sorski, L., Shaashua, L., Melamed, R., Rosenne, E., Page, G. G., & Ben-Eliyahu, S.	2026	Stress impairs the efficacy of immune stimulation by CpG-C: Potential neuroendocrine mediating mechanisms and significance to tumor metastasis and the perioperative period	Wrong type of publication
Livingstone, E, Hollestein, L, van Herkel-Sukel, M, van de Poll-Franse, L, Nijsten, T, Schadendorf, D, de Vries, E	2013	Betablocker use and survival in Dutch melanoma patients with thick primary tumors	Wrong type of publication
Massalee, R., & Cao, X.	2023	Repurposing beta-blockers for combinatory cancer treatment: effects on conventional and immune therapies.	Wrong study design
McCarty, M. F.	2014	A role for cAMP-driven transactivation of EGFR in cancer aggressiveness - Therapeutic implications.	Wrong study design
Nagaraja, A. S., Sadaoui, N. C., Lutgendorf, S. K., Ramondetta, L. M., & Sood, A. K.	2013	β-blockers: A new role in cancer chemotherapy? Expert Opinion on Investigational Drugs	Wrong study design
Nair, S. G., Benny, S., Jose, W. M., & Aneesh, T. P.	2024	Beta-blocker adjunct therapy as a prospective anti-metastatic with cardio-oncologic regulation.	Wrong study design
Pallasch, F. B., & Schumacher, U.	2020	Angiotensin inhibition, TGF-β and EMT in cancer	Wrong study design
Shimizu, A., Kaira, K., Mori, K., Kato, M., Shimizu, K., Yasuda, M., Takahashi, A., Oyama, T., Asao, T., & Ishikawa, O.	2016	Prognostic significance of β2-adrenergic receptor expression in malignant melanoma.	Wrong drug

Stanojkovic, T. P., Zizak, Z., Mihailovic-Stanojevic, N., Petrovic, T., & Juranic, Z. (2005).	2005	Inhibition of proliferation on some neoplastic cell lines-act of carvedilol and captopril.	Not retrieved
Tadic, M., & Grassi, G.	2021	The interactions between antihypertensive drugs and novel anticancer therapy.	Wrong type of publication
Tang, J., Li, Z., Lu, L., & Cho, C. H.	2013	β-Adrenergic system, a backstage manipulator regulating tumour progression and drug target in cancer therapy.	Wrong study design
Unter, S., & Shipman, A.	2016	Angiotensin receptor antagonist treatment, melanoma incidence and ethnicity.	Wrong type of publication
Vaklavas, C., Chatzizisis, Y. S., & Tsimberidou, A. M.	2011	Common cardiovascular medications in cancer therapeutics	Wrong study design
van Beek, N., Dohse, A., Riechert, F., Krull, V., Recke, A., Zillikens, D., & Schmidt, E.	2014	Serum autoantibodies against the dermal-epidermal junction in patients with chronic pruritic disorders, elderly individuals and blood donors prospectively recruited	Wrong population
Vojvodic, A., Vojvodic, P., Vlaskovic-Jovicevic, T., Sijan, G., Dimitrijevic, S., Peric-Hajzler, Z., Matovic, D., Wollina, U., Tirant, M., Thuong, N. van, Fioranelli, M., & Lotti, T.	2019	Beta blockers and melanoma.	Wrong study design
Wang, D. Y., McQuade, J. L., Rai, R. R., Park, J. J., Zhao, S., Ye, F., Beckermann, K. E., Rubinstein, S. M., Johnpulle, R., Long, G. v., Carlino, M. S., Menzies, A. M., Davies, M. A., & Johnson, D. B.	2020	The Impact of Nonsteroidal Anti-Inflammatory Drugs, Beta Blockers, and Metformin on the Efficacy of Anti-PD-1 Therapy in Advanced Melanoma	Wrong population
Wnorowski, A., Sadowska, M., Paul, R. K., Singh, N. S., Boguszewska-Czubara, A., Jimenez, L., Abdelmohsen, K., Toll, L., Jozwiak, K., Bernier, M., & Wainer, I. W.	2015	Activation of β2-adrenergic receptor by (R,R')-4'-methoxy-1-naphthylfenoterol inhibits proliferation and motility of melanoma cells.	Wrong drug

Wrobel LJ, Boehncke WH, Le Gal FA	2018	[Repurposing of betablockers in dermatology].	Wrong study design
Yan, X., Liu, P., Li, D., Hu, R., Tao, M., Zhu, S., Wu, W., Yang, M., & Qu, X.	2022	Novel evidence for the prognostic impact of β-blockers in solid cancer patients receiving immune checkpoint inhibitors	Wrong population
Yang, E. v., & Eubank, T. D.	2013	The impact of adrenergic signaling in skin cancer progression: Possible repurposing of β-blockers for treatment of skin cancer	Wrong study design
Zaenker, K. S.	2012	Beta-adrenergic receptors (β-ARs) expressed at tumor cells: Neuralgic points when targeted by β-blockers to jeopardize metastases formation (from an anthropomorphic view of a tumor cell).	Wrong study design
Zhang, X., Liao, Y.-L., Sun, Y., & Sun, W.-J.	2011	Inhibitory effect of amlodipine on spontaneous pulmonary metastasis of murine melanoma B16 cells and relevant mechanism.	Not retrieved
Zhou, X., Jiao, L., Qian, Y., Dong, Q., Sun, Y., Zheng, W. v., Zhao, W., Zhai, W., Qiu, L., Wu, Y., Wang, H., Gao, Y., & Chen, J.	2021	Repositioning azelnidipine as a dual inhibitor targeting cd47/sirpα and tigit/pvr pathways for cancer immuno-therapy	Wrong population
-	2017	Fewer relapses of melanoma with propranolol: A new area of application for an "old" beta blocker?	Wrong type of publication
De Moura et. al.	2025	Survival disadvantage with beta-blocker use in melanoma brain metastases	Not retrieved
Selvaraj, K.J. et al.	2025	A randomised pilot trial of perioperative propranolol combined with celecoxib versus standard of care in stage III melanoma: The ProCel study protocol	Wrong type of publication
De Giorgi, V. et al.	2013	Effect of β-blockers and other antihypertensive drugs on the risk of melanoma recurrence and death	Wrong study design

De Giorgi, V. et al.	2018	Propranolol for Off-label Treatment of Patients With Melanoma: Results From a Cohort Study.	Wrong study design
De Giorgi, V. et al.	2011	Propranolol for Off-label Treatment of Patients With Melanoma: Results From a Cohort Study.	Wrong study design
Drobni, Z et al.	2022	Renin–angiotensin–aldosterone system inhibitors and survival in patients with hypertension treated with immune checkpoint inhibitors.	Wrong study design
Everatt, R. et al.	2025	Postdiagnostic use of antihypertensive medications and survival in colorectal, lung, corpus uteri, melanoma and kidney cancer patients with hypertension.	Wrong study design
Gandhi, S. et al.	2021	Phase I Clinical Trial of Combination Propranolol and Pembrolizumab in Locally Advanced and Metastatic Melanoma: Safety, Tolerability, and Preliminary Evidence of Antitumor Activity	Wrong study design
Katsarelias, D. et al.	2020	The effect of beta-adrenergic blocking agents in cutaneous melanoma—a nation-wide swedish population-based retrospective register study	Wrong study design
Kennedy, O. J. et al.	2022	Prognostic and predictive value of β-blockers in the EORTC 1325/KEYNOTE-054 phase III trial of pembrolizumab versus placebo in resected high-risk stage III melanoma	Wrong study design
Livingstone, E. et al.	2013	β-Blocker use and all-cause mortality of melanoma patients: Results from a population-based Dutch cohort study	Wrong study design
McCourt, C. et al.	2014	Beta-blocker usage after malignant melanoma diagnosis and survival: a population-based nested case-control study	Wrong study design
Wrobel, L. J., Gayet-Ageron, A., & Gal, F. A.	2020	Effects of Beta-Blockers on Melanoma Microenvironment and Disease Survival in Human	Wrong study design
